# Superficial Retinal Microvasculature and Choriocapillaris Alterations after Photodynamic Therapy in Chronic Central Serous Chorioretinopathy

**DOI:** 10.1155/2022/4024603

**Published:** 2022-07-31

**Authors:** Morteza Entezari, Iman Ansari, Alireza Ramezani, Humayoun Nikkhah, Parisa Mohammadi, Bahar Kheiri

**Affiliations:** ^1^Negah Aref Ophthalmic Research Centre, Shahid Beheshti Medical Sciences, Tehran, Iran; ^2^Ophthalmic Research Center, Department of Ophthalmology, Imam Hossein Medical Center, Shahid Beheshti Medical Sciences, Tehran, Iran

## Abstract

**Purpose:**

To compare superficial retinal vascular, choriocapillaris (CC), and choroidal thickness changes in chronic central serous chorioretinopathy (CSCR) patients after half-dose photodynamic therapy (PDT).

**Method:**

In this prospective interventional case series study, fifteen eyes of 14 patients with chronic CSCR undergoing half-dose PDT treatment were enrolled. All patients underwent enhanced depth imaging optical coherence tomography angiography (EDI-OCT) and optical coherence tomography angiography (OCTA) at baseline and 3 months after treatment. Best-corrected visual acuity (BCVA), superficial retinal vascular density, CC vessel density, foveal avascular zone (FAZ), central macular thickness (CMT), and choroidal thickness were compared.

**Results:**

Mean BCVA before and after PDT was 0.34 ± 0.26 and 0.19 ± 0.25 logMAR, respectively (*p*=0.011). Mean FAZ before treatment was 410.21 ± 117.00 *μ*m^2^, which increased to 433.50 ± 116.76 *μ*m^2^ (*P*=0.253). Mean vessel density at superficial capillary plexus (SCP) was 38.93 ± 11.12 at baseline, which increased to 39.04 ± 11.43 (*P*=0.886). Mean CC vessel density was 53.21 ± 4.14 at baseline, which significantly decreased to 51.85 ± 4.21 (*P*=<0.001). BCVA has no significant correlation with FAZ (*P*=0.282) and vessel density (*P*=0.0.241) at SCP. CMT significantly decreased from 380.87 ± 41.66 *μ* at baseline to 268.20 ± 28.07 *μ* at 3 months (*P*=0.132). We did not find any correlation between CMT and FAZ (*P*=0.040) and vessel density (*P*=0.686) at SCP. Mean subfoveal choroidal thickness reduced from 411 ± 171 *μ*m before treatment to 372 ± 117 *μ*m (*P*=0.106).

**Conclusion:**

PDT treatment can affect retinal and choroidal structural parameters, but we could not find any significant influence on retinal vascular parameters, including FAZ area and vessel densityonly mean CC vessel density and subfoveal choroidal thickness decreased.

## 1. Introduction

Central serous chorioretinopathy (CSCR) is one of the vision-threatening retinopathies affecting young males, but it can involve both males and females with older age. The incidence of CSCR is 9.9 and 1.7 per 100 000 individuals in males and females, respectively [1, 2]. Acute CSCR is described by an accumulation of subretinal fluid (SRF) under the neurosensory retina and pigment epithelium detachment (PED) [3]. Although acute CSCR is often self-resolving, in some cases, it becomes chronic and permanent, causing retinal pigment epithelium (RPE) and photoreceptor alterations with a progressive visual loss over a period of 3–6 months [4]. Photodynamic therapy (PDT) was first used in the treatment of CSCR by Yannuzzi, and then in various studies, it has been shown that PDT may be the most effective treatment in reducing retinal and subretinal fluid, improving overall vision, and rapid vision improvement [5, 6].

Enhanced depth imaging optical coherence tomography (EDI-OCT), introduced by Spaide, provides more detailed tomographic images of the outer layers of the globe, particularly the choroid. More studies reported that the choroidal thickness in eyes with CSCR was significantly greater than normal eyes, as shown on EDI-OCT. Some studies showed that choroidal thickness decreased after treatment [7, 8].

Optical coherence tomography angiography (OCTA) is a new technology that enables rapid, non-invasive, dye-free visualization of the vascularization of retinal and choroidal vessels [9]. Although there are studies that have highlighted the qualitative and structural changes in CSCR by OCTA for finding CSCR complications such as CNV [4], using OCTA showed that CC vessel density was significantly reduced in CSCR patients after half-dose PDT [10].

In previous studies, it has been revealed that the pathological changes of CSCR are mainly choroidal microvascular abnormalities, and whether there is a change in the microvessels in the macular region remains unexplored. Recently, in one study, they evaluated the changes of retinal microvascular network in patients with CSCR, and they found retinal superficial microvessel density reduction in the macular regions of CSCR [11]. There are a few studies about quantitative changes after treatment with PDT in CSCR patients [4].

In this study, we evaluated superficial retinal vascular, CC vessel density, and choroidal thickness changes in patients with CSCR after half-dose PDT by OCTA and EDI-OCT.

## 2. Method

In this prospective interventional case series study, fifteen eyes of 14 patients with chronic CSCR who underwent half-dose PDT at the Negah Eye Hospital from September 2019 to December 2020 were enrolled. The study protocol was approved by the local ethics committee and adhered to the tenets of the Declaration of Helsinki. All patients signed a written general consent to participate in clinical studies.

Inclusion criteria included the following: patients with a diagnosis of chronic CSCR (more than 3 months); age ≥18 years old; no previous ocular surgery; any prior PDT or laser therapy. Exclusion criteria were as follows: history of diabetes and hypertension; pregnancy and lactation; presence of CNV and any other retinal disease; vitreoretinal disease or retinal surgery; any other systemic and retinal disease that has impaired retinal and choroidal circulation; any media haziness that causes interruption of appropriate image quality and high myopia ≥6.

**Figure 1:**
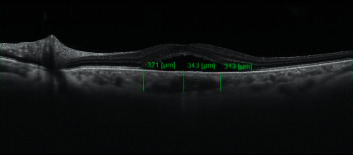
Enhanced-depth imaging (EDI)—OCT demonstrates subfoveal choroidal thickness (SFCT) and choroidal thickness 1500 micrometers temporal and nasal to the fovea.

Demographic data and medical history of enrolled patients were recorded in the questionnaire charts. All the patients underwent a complete ophthalmic examination at baseline and 3 months after treatment including best-corrected visual acuity (BCVA), slit lamp examination, intraocular pressure (IOP) measurement, and dilated fundus examination by indirect ophthalmoscope and +78 non-contact lens. All cases underwent structural spectral-domain (SD-OCT) (HRA Heidelberg, Heidelberg, Germany) and 3 × 3 mm OCTA (Topcon DRI OCT Triton, Topcon Corporation, Japan) scans of the macula, enhanced depth imaging optical coherence tomography angiography (EDI-OCT) fluorescein and Indocyanine green angiography(HRA Heidelberg, Heidelberg, Germany). OCTA and EDI-OCT were repeated after 3 months. EDI-OCT was obtained by Spectralis OCT at baseline and 3 months after treatment. Subfoveal choroidal thickness (SFCT) and 1500 *μ* temporal and nasally measured vertically from the outer border of the RPE to the inner border of the sclera in micrometers (Figure 1).

**Figure 2:**
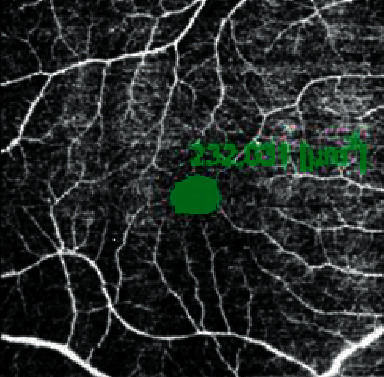
The fovea avascular zone (FAZ) area has been manually plotted using the polygon selection tool in the superficial capillary plexus (SCP) and its dimension is reported in square micrometers. The end tip vessels at SCP were manually marked and these areas were connected and the area of the central area was calculated by the device.

**Figure 3:**
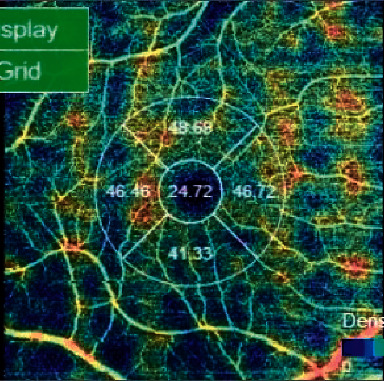
Vessel density at SCP was automatically calculated by the device and reported as a percentage in the central area and four quadrants (superior, nasal, temporal, and inferior). Areas with blood vessels and flow are shown in warm colors (red) and areas without blood vessels are shown in cold colors (blue).

The minimum strength of OCTA images was 7 out of 10. In order to evaluate the angiographic images, the superficial capillary plexus (SCP) was automatically segmented and manually adjusted in case of segmentation errors by a single operator. Choriocapillaris was segmented with an inner boundary 30 *μ*m below the RPE-Bruch's membrane complex and an outer boundary 60 *μ*m below the RPE-Bruch's membrane complex. The fovea avascular zone (FAZ) area has been manually plotted using the polygon selection tool in SCP and its dimension in square micrometers has been calculated (Figure 2). Vessel density at SCP and choriocapillaris layer was automatically calculated by the device and reported as a percentage in the central area and four quadrants (Figure 3).

PDT with half-dose and full-fluence rate (50 J/cm^2^) was done by one single experienced physician. Verteporfin (Visudyne; Novartis Pharma, New York, NY, USA) 3 mg/m^2^ was infused for 10 min, and 5 min after the start of the infusion, a laser light at 689 nm was delivered at 50 J/cm^2^ with an intensity of 600 mW/cm^2^ for 83 seconds.

### 2.1. Statistical Analysis

Age, sex, eye, BCVA, central macular thickness (CMT), SFCT, FAZ area, and vessel density at SCP were included in the analysis. The collected data were entered into SPSS software (IBM Corp. Released 2014. IBM SPSS Statistics for Windows, Version 23.0. Armonk, NY: IBM Corp.); normal continuous variables were reported using mean and standard deviation; qualitative variables were reported as frequency and percentage. Variable change during the follow-up period was assessed with analysis of variance (ANOVA) for repeated measures, and the Tukey test was used as post hoc test. The relationship between variables was explored using Pearson correlation test and multiple correlations were adjusted by means of Benjamini–Hochberg test. A *P* value of less than 0.05 was considered statistically significant.

## 3. Results

Fifteen eyes of 14 consecutive patients were analyzed. 9 (65%) of patients were male. The mean age of patients was 49.7 ± 10.24 (range 33–70). The left eye was involved in 11 (73%) of cases. Mean duration from diagnosis to treatment was 19 ± 20.19 months (range 3–60). One patient received treatment bilaterally (Table 1).

The mean BCVA before and 3 months after PDT was 0.34 ± 0.26 logMAR and 0.19 ± 0.25 logMAR, respectively. The change in BCVA was statistically significant (*P*=0.011). There was no significant correlation between BCVA and structural parameters including CMT (*P*=0.23) and SFCT (*P*=0.439). In evaluation of the relationship between BCVA and OCTA findings, we did not find any significant correlation between BCVA with FAZ (*P*=0.282) and vessel density (*P*=0.0.241) at SCP.

CMT decreased from 380.87 ± 41.66 *μ* at baseline to 268.20 ± 28.07 *μ* at 3 months. This CMT reduction was not statistically significant (*P*=0.132). After 3 months of follow-up, there is no significant correlation between CMT with FAZ area (*P*=0.040) and vessel density (*P*=0.686) at SCP, respectively.

**Figure 4:**
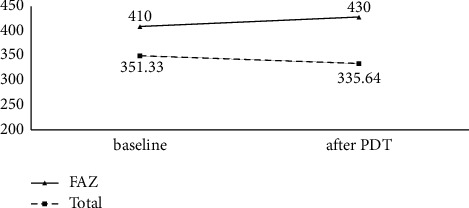
Comparison of FAZ and total choroidal thickness before and three months after treatment.

Mean SFCT decreased from 411 ± 171 *μ* at baseline to 372 ± 117 *μ* 3 months after PDT. The decrease in SFCT at 3 months after PDT treatment compared to baseline was not statistically significant (*P*=0.106). Comparing the SFCT and OCTA parameters, there was no significant correlation between SFCT with FAZ area (*P*=0.406) and vessel density (*P*=0.055) at SCP, respectively (Table 2) (Figure 4).

Mean FAZ area at SCP before treatment was 410 ± 117 *μ*m^2^ and increased to 430 ± 117 *μ*m^2^ after PDT. The change in FAZ area from baseline was not statistically significant (*P*=0.253). Mean vessel density at SCP was 38.93 ± 11.12 at baseline and increased to 39.04 ± 11.43 following treatment (*P*=0.886), and mean CC vessel density was 53.21 ± 4.14 at baseline and significantly decreased to 51.85 ± 4.21 after treatment (*P*=<0.001).  Table 3 shows the mean vessel density of the other quadrants at the SCP and choriocapillaris layer before and three months after PDT treatment.

## 4. Discussion

In our study, by using OCTA and structural OCT, we investigated quantitative retinal and choroidal changes in patients with chronic CSCR before and three months after PDT treatment. Our study revealed no significant relationship between BCVA and structural variables (CMT, SFCT) with OCTA parameters (FAZ area, vessel density) at SCP. Because CSCR is a choroidal and outer retinal disease and the inner retina is not affected in this disease, the FAZ area and vascular density at the SCP have not been affected in our study [4]. Our finding was consistent with Rabiolo et al research, but another study by Yu et al, which highlighted retinal superficial microvessel density reduction in the macular area of patients with CSCR was different from our finding [4, 11]. Our study concluded that CC vessel decreased significantly after 3 months of half-dose PDT treatment. In other study, Nassisi et al. showed that CC vessel density was significantly reduced at 1 week as compared with baseline, suggesting a possible short-term effect of PDT on CC perfusion. After 1 month, however, the CC vessel density was even higher than the baseline, probably due to a CC recovery [10].

In a case control study, Yu et al. used OCTA to compare macular microvascular, macrovascular and total vessel density at SCP. The results of this study showed that total macular retinal microvessel density was significantly reduced in CSCR patients compared with the control group, suggesting that CSCR induces changes of choroidal capillary and may also cause the change of retinal capillaries, but the mechanism needs further study [11]. One of the findings of the study was the reduction in macular retinal vascular density near central fovea area. However, the comparison between patients with CSCR and the control group showed that this decrease in macular retinal vascular density was not statistically significant, and probably due to serous macular detachment in CSCR, no correlation between macular region and vascular density was observed in these patients [11]. This study suggested that the reduction of retinal vascular network in CSCR patients can be an indicator of the progression of macular degeneration, which is usually manifested as retinal vascular disease [11].

CSCR is considered part of the pachychoroid disease spectrum [12]. Dilated and congested choroidal vessels (pachyvessel) increased choroid thickness and leakage through RPE resulting in subretinal fluid accumulation and neurosensory detachment [13, 14]. It is believed that the role of PDT in the treatment of CSCR is through the production of free radicals and damage to the vascular endothelium, resulting in thrombosis and obstruction of dilated choroidal vessels [15, 16]. Therefore, PDT reduces the choroidal thickness by targeting the choroidal large vessels [14]. Many previous studies have shown the reduction of subfoveal choroidal thickness following PDT treatment, and this reduction is seen following different PDT protocols. Half-dose, full-fluence PDT has been reported to be as effective as half-fluence, full-dose PDT [17–19]. According to reports, in our study, following the half-dose full-fluence PDT, SFCT, and choroidal thickness 1500 micrometers to the nasal and temporal from fovea was reduced, which was more pronounced in the SFCT.

Our study had some limitation including the small sample size, short follow-up period, and evaluation of OCTA parameters only at SCP. Therefore, studies with longer follow-up period, more sample size, and evaluation of other retinal layers are needed to determine the role of retinal vessels in CSCR.

## 5. Conclusion

PDT treatment, in addition to retinal and choroidal structural parameters, can affect retinal vascular parameters, including FAZ area and vessel density, although these changes are not very pronounced in the superficial layers of the retina.

## Figures and Tables

**Table 1:** Demographic data.

Patient number Eye		14 15
Sex	Male	9 (65%) 5 (35%)
	Female	
Age		49.7 ± 10.24 (range 33–70)
Eye	Right	**4 (27%)** **11 (73%)**
	Left	
Duration from diagnosis to treatment		19 ± 20.19 months (range 3–60)

**Table 2:** Subfoveal choroidal thickness (SFCT) and choroidal thickness 1500 micrometers temporal and nasal to the fovea.

	Mean	Standard deviation	*P* value
Subfoveal (baseline) Subfoveal (after PDT)	411.07 372.73	171.80 117.55	0.106

Temporal (baseline) Temporal (after PDT)	315.00	109.72	0.777
	310.60	103.87	

Nasal (baseline) Nasal (after PDT)	327.93	102.31	0.797
	323.60	102.56	

Total (baseline) Total (after PDT)	351.33	135.66	0.145
	335.64	109.12	

**Table 3:** Mean vessel density of the quadrants at the SCP and choriocapillaris before and three months after PDT treatment.

	SCP			Choriocapillaris		
	Mean	Standard deviation	*P* value	Mean	Standard deviation	*P* value
Central (baseline) Central (after PDT)	19.17 18.27	5.06 4.87	0.511	47.53 46.40	3.75 4.15	0.141

Superior (baseline) Superior (after PDT)	45.84 46.14	5.02 5.99	0.879	53.70 52.19	2.45 3.37	0.184

Temporal (baseline) Temporal (after PDT)	45.74 45.63	3.04 3.46	0.935	55.87 54.69	3.24 2.74	0.007

Inferior (baseline) Inferior (after PDT)	40.67 41.37	4.58 3.79	0.655	53.95 52.48	2.41 2.50	0.002

Nasal (baseline) Nasal (after PDT)	43.28 43.79	5.60 3.63	0.775	54.99 53.48	2.79 2.79	<0.001

Total (baseline) Total (after PDT)	38.93 39.04	11.12 11.43	0.886	53.21 51.85	4.14 4.21	<0.001

## Data Availability

The data used to support the findings of this study are included within the article.
